# Maintenance of smoking cessation in Korean single mothers

**DOI:** 10.1186/s12905-021-01426-x

**Published:** 2021-08-09

**Authors:** Mi-Ji Lee, Kang-Sook Lee

**Affiliations:** 1 Graduate School of Public Health, The Catholic University of Korea, Seoul, Republic of Korea; 2 Seoul Tobacco Control Center, Seoul, Republic of Korea; 3 Department of Preventive Medicine, College of Medicine, The Catholic University of Korea, 222 Banpo-daero, Seocho-gu, Seoul, 06591 Republic of Korea

**Keywords:** Single mothers, Smoking cessation, Smoking during pregnancy, Smoking cessation counseling program

## Abstract

**Background:**

Single mothers in South Korea are vulnerable to developing smoking habits, due to many difficulties and limitations; however, they have often been overlooked by smoking cessation support services. Therefore, this study aimed to investigate the general and smoking-related characteristics of single mothers registered with the Visiting a Smoking Cessation Service in Seoul, South Korea, to identify factors associated with smoking cessation maintenance at 4 weeks and 24 weeks after they initially quit smoking.

**Methods:**

The participants were 77 single mothers registered in the Smoking Cessation Service Program. Data were included from a three-year span (January 2017–December 2019). Smoking cessation counseling, motivational enhancement, and self-exploration counseling were provided for six months. The participants were evaluated on their smoking cessation status at 4 weeks and 24 weeks.

**Results:**

Most participants were aged 22 years or younger. The rates of smoking cessation maintenance were 58.4 and 18% at 4 weeks and 24 weeks, respectively. The higher the number of counseling sessions, the higher the participants’ chances of maintaining smoking in all non-smoking periods, and whether pregnancy, CO level, and drinking were significant only in a short-term non-smoking period (4 weeks).

**Conclusions:**

Our results suggest that the number of smoking cessation counseling sessions is important for long-term smoking cessation beyond short-term cessation in single mothers. To increase the smoking cessation rate of single mothers, it is important to conduct customized smoking cessation counseling at the time of smoking cessation and continue such counseling in the long term.

## Background

The South Korean family structure has undergone drastic changes due to social and cultural shifts, resulting in delayed marriage or pregnancy and increased divorce or separation. Thus, more individuals are living alone or within atypical families [1, 2]. This change resulted in unmarried parents, especially 20,761 single mothers and 7082 single fathers in Korea in 2019, with single mothers accounting for 74.5% of all single parents [3].

Several studies have shown that the smoking rate is higher among single mothers than among married mothers [4, 5]. This phenomenon can be attributed to the societal experiences that single mothers have following childbirth, which are considerably different from those of married mothers. Single mothers experience high levels of physical and mental stress as they raise a child on their own [6]. They are consistently exposed to higher levels of stress resulting from socioeconomic burdens, as compared to married mothers [7]. Single mothers are more likely to suffer from depression than married mothers [8, 9]. The incidence of mental health disorders is also higher among young single mothers, as they have a short amount of time to adapt to society and tend to have children who require high levels of parental responsibility [10].

Further, single mothers are not only vulnerable to mental health disorders but also to physical health problems. For instance, they are at higher risk than married mothers for conditions such as hypertension, obesity, and diabetes [11]. Single mothers are often forced to discontinue their education or careers to care for their children, which can lead to financial difficulties. Moreover, the younger a single mother is, the greater the financial burden she faces [10, 12]. Financial difficulties can seriously disrupt a child’s social, emotional, and intellectual development [13, 14].

The difficulties that single mothers face can lead to the abuse of substances such as alcohol and nicotine [15], and single mothers are more likely to smoke cigarettes than married mothers [4, 9, 16]. Without effective strategies for coping with difficult situations, individuals often resort to smoking to manage stress. A study on women with low income found that they tended to smoke to control their mood in stressful situations [17]. Notably, smoking not only affects a single mother’s health but also that of her fetus or child. As younger people are generally more affected by smoking, and single mothers tend to give birth at younger ages than married mothers, single mothers and their infants are typically more vulnerable to the risks of smoking [18]. It is well established that smoking during pregnancy increases the prevalence of premature birth, low birth weight, and sudden infant death syndrome [19]. Pregnancy is a strong reason for both temporary and long-term smoking cessation. Smoking relapses often occur during pregnancy, and single mothers are more likely to relapse than married mothers [20].

Smoking among single mothers is a high-priority public health concern, and healthcare services must be actively provided to reduce the rate of smoking among single mothers because smoking is a major preventable risk factor [21]. Despite the need for smoking cessation programs tailored to single mothers, research on systematic health promotion programs for single mothers is lacking. This study aimed to investigate smoking-related characteristics and factors associated with smoking cessation maintenance among single mothers by providing smoking cessation counseling services tailored to this population.

## Methods

### Participants

Data were collected from participants registered in the Smoking Cessation Service Integrated Information System (https://www.nosmk.khealth.or.kr) from January 1, 2017, to December 31, 2019. The participants were single women who were pregnant or had given birth and resided in a facility for single mothers at the time of service registration. In our study, there were no exclusion criteria, as we investigated all single mothers who quit smoking from two institutions, Aeranwon and Dodam House. Single mothers admitted to single mothers’ facilities are not allowed to smoke within the facility, but may smoke when they go out. Smoking prevention education and smoking cessation are recommended to provide a variety of smoking cessation services, but smoking cessation is not coercive. Single mothers who smoke participated in this study on the recommendation of the institutional staff or of voluntary smoking cessation services. Therefore, failure to participate did not have adverse consequences. We determined the minimum sample size required for t-test using G*POWER version 3.0 [22], effect size = 0.8, α = 0.05, 1 − β = 80%, which was calculated to be 42 people. In total, 77 participants were selected in consideration of the drop-out rate. This study was approved by the Institutional Review Board of the Catholic University of Korea (MC20ZESI0011).

### Measurement

#### General characteristics

The following general characteristics were examined: age, pregnancy status, drinking, exercise, and education level. Drinking was classified as either “yes” or “no,” depending on whether the participant had consumed alcohol in the last year. Exercise was classified as either “yes” or “no,” depending on whether a participant engaged in at least 10 min of moderate-intensity exercise once per week [23].

### Smoking related factors

Smoking-related factors included exhaled carbon monoxide (CO) level, number of counseling sessions attended, nicotine dependence, and scores on the “importance,” “confidence” and “readiness” motivation rulers for smoking cessation.

Nicotine dependence was measured using the Fagerström Test (FTND), in which items are scored from to 0−10 points, with higher scores indicating higher levels of nicotine dependence. According to FTND score, nicotine dependence can be classified as low (0– 3 points), moderate (4−6 points), or high (7−10 points) [24]. Three motivation rulers were used to assess participants’ motivation for smoking cessation. Importance was assessed using the following item, “How important is stopping smoking to you?” (0 = Not important at all; 10 = Most important goal of my life). Confidence was assessed by, “How confident are you that you will quit smoking within the next month?” (0 = Not at all; 10 = 100% confident). Readiness was assessed by, “How ready are you to quit smoking within the next month?” (0 = Not at all; 10 = 100% ready) [25, 26].

### Smoking cessation supporters

To know whether they had support from others in their smoking cessation efforts, participants were asked the question, “Who helps you quit smoking?” Ten groups were identified as supporters for smoking cessation: parents/grandparents, siblings, partners, children, friends/school peers, coworkers, teachers, healthcare professionals, others, and no one.

### Reasons for smoking cessation

Participants were asked to list one reason why they wanted to quit smoking. Nine possible responses were provided: “advice from family or others around me,” “for my health,” “financial reasons,” “environmental reasons such as the expansion of non-smoking areas,” “to have a clean image,” “for the health of those around me,” “to show my willingness to cease smoking,” “due to society’s view on smokers,” and “others.”

### Counseling process

A smoking cessation counselor, who was also a member of the current research team, created an environment conducive to smoking cessation within a single mother facility located in Seoul. Throughout the research period, the counselor provided smoking cessation education to encourage voluntary participation in smoking cessation counseling. The smoking cessation education program informed the participants of the effects of smoking on mothers and fetuses/infants using visual and auditory media, such as images and videos. The program also taught participants why smoking is more lethal to women and infants than to men and the harmful effects of secondhand smoke. Prior to counseling, all participants provided informed consent and gave their signatures.

The participants attended approximately nine tailored smoking cessation counseling sessions over six months after registering for the Smoking Cessation Service Program. The consultation session comprised nine sessions, based on the “END program” [27] developed by the Korea Health Promotion Institution and the smoking cessation counseling manual of the health center, in accordance with the Guidelines for the National Smoking Treatment Projects. A smoking cessation counselor visited the single mother facility to provide counseling. If face-to-face counseling was not possible, a telephone interview was conducted. Exhaled CO level, lung volume, and blood pressure were measured at each counseling session to assess their physical condition. The success of smoking cessation was determined based on whether exhaled CO level was measured at 6 ppm or below and cotinine levels. Customized smoking counseling for single mothers is based on behavioral theories such as the Stages of Change Model, Social Influence Model, Social Cognitive Theory, Health Belief Model, and Theory of Planned Behavior. During the tailored counseling program, the participants took part in activities including examining their stress levels using a machine that measures heart rate variability (HRV), self-efficacy score, strengths, values, drawing a tree for future plans, and looking back on achievements, while gaining knowledge on smoking cessation. In particular, almost all sessions of the program focused on psychological, social, and economic stress in the pregnancy and parenting process that is experienced only by single mothers, allowing them to find and apply realistic countermeasures [28]. We aimed to understand the stress situation of single mothers and recognize their feelings about their situations. We then let the participants share what they can and cannot do about the situation and helped them apply coping measures by using their internal strengths. The program also helped single mothers determine from which institutions to seek help and receive social support. Thereafter, the coping measures were evaluated, supplemented, and continued so that participants could fully adopt the measures by the end of the consultation. In the last session, a congratulatory ceremony was held among the participants who maintained smoking cessation for 24 weeks (Table 1). The smoking cessation counselor reviewed the goals and specific details of participants from each session of the program and became familiar with the consultation details through demonstrations. After the consultation began, a case conference was held quarterly to receive clinical supervision. Counselors prepared counseling logs for each case and received observations, evaluations, and feedback from the supervisor to provide safe and appropriate services to single mothers.

**Table 1:** Manual for counseling

Session	Schedule	Topics
1	Enrollment (Day 1)	Quitting smoking education (the harmful effects of smoking in women and pregnant women, indirect smoking in fetuses and infants)
		Introduction and guidance of programs
		Providing information on consent & Writing registration card
		Assessment of smoking history and smoking behavior
		Providing Fagerström Test of Nicotine Dependence (FTND)
		Determine to quit smoking and establish the start day
		Measuring exhaled carbon monoxide
		Check the stress situation
2	Abstinence at 2-week (Day 14)	Measuring exhaled carbon monoxide
		Confirm whether participants began quitting smoking
		Self-esteem test and psychological test
		Finding My Strength
		Check my values
		Check the stress situation & Understand the situation
3	Abstinence at 4-week (Day 28)	Measuring exhaled carbon monoxide & Check maintenance of abstinence
		Check for withdrawal symptoms
		Learn how to deal with withdrawal symptoms
		Check the stress situation & Take action
4	Abstinence at 6-week (Day 42)	Measuring exhaled carbon monoxide & Check maintenance of abstinence
		My first experience of smoking and history of smoking
		Check the amount of smoking by converting it into money
		Examine the benefits of quitting
5	Abstinence at 8-week (Day 56)	Measuring exhaled carbon monoxide
		Think of a situation where it's hard for me to refuse cigarettes
		Practicing Refusing Tobacco Through a Situation Play
		Planning to replace smoking habits
		Coping with Stress
6	Abstinence at 12-week (Day 84)	Measuring exhaled carbon monoxide & Check maintenance of abstinence
		Knowing the dangers of e-cigarettes and drug addiction
		Coping with Stress
7	Abstinence at 16-week (Day 112)	Measuring exhaled carbon monoxide
		Check for withdrawal symptoms
		Check my change of body after quitting smoking
		Making My Own quitting smoking Prescriptions
		Assessment of stress Coping
8	Abstinence at 20-week (Day 140)	Measuring exhaled carbon monoxide
		Learn the rules for preventing re-smoking
		Drawing my future plans tree (My Past, Present, and Future)
		Writing down my experience of achievement
9	Abstinence at 24-week (Day 168)	Validation of abstinence at 6-month (measuring exhaled carbon monoxide/urine cotinine test)
		Guidance on further consultation
		Commemorating the success of 6-month smoking cessation
		Providing a souvenir for a successful quitting

### Statistical analysis

IBM SPSS® software version 26 was used to perform statistical analyses. To examine the differences in the rates of smoking cessation at 4 weeks and 24 weeks according to participants’ general and smoking-related characteristics, the categorical data were compared using a chi-square test, and continuous data using a t-test. We used logistic regression model adjusted by age and education level for pregnancy, drinking, CO level, and number of counseling sessions which were in significant differences between smoking and abstinence group. All *p* values were set at < 0.05.

## Results

### General characteristics of the participants

Of the 77 single mothers who participated in the program, 47.0% were aged 20 years or younger. During the study, 29.0% of the single mothers were pregnant and 35.0% were single mothers who drank. The average CO level for single mothers was 3.92 ppm, and the average number of counseling sessions was 4.17. Low level of Nicotine dependence was 73.0%. Regarding motivation rulers, importance in the 8–10 range was 49.0%, confidence in the 4–7 range was 49.0%, and readiness in the 4–7 range was 38.0%. The most common reason participants provided for wanting to quit smoking was “for my health” (24.0%), followed by “other” reasons, “advice from family or others around me,” “for the health of those around me,” “financial reasons,” “to have a clean image,” “environmental reasons such as the expansion of non-smoking areas,” “to show my willingness to cease smoking,” and “due to society’s view on smokers.” Of all the participants, 22.0% reported that they had no one to support their smoking cessation efforts. The most commonly identified source of support for participants’ smoking cessation efforts was parents/grandparents, followed by teachers, friends/school peers, others, children, and siblings (Table 2).

**Table 2:** General characteristics of the study subjects

Variable	N = 77(100%) or M ± SD
Age	
≤ 20	36 (47.0)
21–25	27 (35.0)
> 25	14 (18.0)
Education level	
Unknown	15 (19.5)
≤ 9y	15 (19.5)
10–12y	37 (48.0)
> 12y	10 (13.0)
Pregnancy	
No	55 (71.0)
Yes	22 (29.0)
Drinking	
No	50 (65.0)
Yes	27 (35.0)
Exercise	
No	56 (73.0)
Yes	21 (27.0)
CO level (ppm)	
M ± SD	3.92 ± 5.07
Number of counseling	
M ± SD	4.17 ± 2.61
Nicotine dependence	
Low	56 (73.0)
Mild	14 (18.0)
High	7 (9.0)
Motivation rulers/ Importance	
0–3	10 (13.0)
4–7	29 (38.0)
8–10	38 (49.0)
Motivation rulers/ Confidence	
0–3	14 (18.0)
4–7	38 (49.0)
8–10	25 (32.0)
Motivation rulers/ Readiness	
0–3	20 (26.0)
4–7	29 (38.0)
8–10	28 (36.0)
The reasons for smoking cessation	
Recommendations from family members or others	11 (14.3)
For your own health	31 (40.3)
Economic reasons	4 (5.2)
Environmental reasons	1 (1.3)
For clean image management	3 (3.9)
For the health of the people around	9 (11.7)
To show willingness to smoking abstinence	1 (1.3)
Because of social gaze	1 (1.3)
Etc	16 (20.8)
The supporters for smoking cessation	
Parents/grandparents	16 (20.8)
Siblings	1 (1.3)
Partner	6 (7.8)
Children	5 (6.5)
Friends/school peers	9 (11.7)
Coworkers	0 (0.0)
Teachers	15 (19.5)
Healthcare professionals	0 (0.0)
Others	8 (10.4)
No one	17 (22.1)

### Maintenance of smoking cessation according to general and smoking related characteristics

The rate of smoking cessation maintenance at 24 weeks was significantly higher among older mothers. In total 15.6 and 27.0% of the pregnant participants responded that they smoked during pregnancy at 4 weeks and 24 weeks, respectively. The rate of smoking cessation maintenance at 4 weeks was significantly higher among pregnant mothers. The rate of smoking cessation maintenance at 4 weeks was significantly higher among those who did not consume alcohol compared to those who did. Significant differences in exhaled CO level were found at 4 weeks between participants who smoked with scores of 6 ppm and those who maintained smoking cessation with scores of 2 ppm; furthermore, participants with low exhaled CO levels had a higher rate of smoking cessation. A significant difference in the number of counseling sessions attended was found between the participants who maintained smoking cessation and those who smoked, with the former attending at least three more counseling sessions than the latter. The rate of smoking cessation maintenance was significantly higher for participants with high scores on the readiness ruler at 4 weeks. There were no significant differences in education levels, nicotine dependence, motivation rulers importance and confidence, reasons for smoking, and smoking cessation supporters (Table 3).

**Table 3:** Maintenance of smoking cessation according to general and smoking related characteristics

Variable	Smoking abstinence					
N (%) or M ± SD	4 weeks			24 weeks		
	Smoking N = 32 (41.6%)	Abstinence N = 45 (58.4%)	*p* value	Smoking N = 63 (81.8%)	Abstinence N = 14 (18.2%)	*p* value
Age						
> 25	16 (50.0)	20 (44.4)	0.061	32 (50.8)	4 (28.6)	0.020
≤ 20	14 (43.8)	13 (28.9)		23 (36.5)	4 (28.6)	
21–25	2 (6.3)	12 (26.7)		8 (12.7)	6 (42.9)	
Education level						
Unknown	8 (25.0)	7 (15.6)	0.600	14 (22.2)	1 (7.1)	0.198
≤ 9y	5 (15.6)	10 (22.2)		12 (19.0)	3 (21.4)	
10–12y	16 (50.0)	21 (46.7)		31 (49.2)	6 (42.9)	
> 12y	3 (9.4)	7 (15.6)		6 (9.5)	4 (28.6)	
Pregnancy						
No	27 (84.4)	28 (62.2)	0.034	46 (73.0)	9 (64.3)	0.513
Yes	5 (15.6)	17 (37.8)		17 (27.0)	5 (35.7)	
Drinking						
No	14 (43.8)	36 (80.0)	0.001	40 (63.5)	10 (71.4)	0.573
Yes	18 (56.3)	9 (20.0)		23 (36.5)	4 (28.6)	
Exercise						
No	26 (81.3)	39 (66.7)	0.157	44 (69.8)	12 (85.7)	0.228
Yes	6 (18.8)	15 (33.3)		19 (30.2)	2 (14.3)	
CO level (ppm)						
M ± SD	6.25 ± 6.47	2.27 ± 2.84	0.002	4.24 ± 5.35	2.50 ± 3.28	0.248
Number of counseling						
M ± SD	2.59 ± 1.74	5.29 ± 2.56	0.000	3.30 ± 1.95	8.07 ± 1.27	0.000
Nicotine dependence						
Low	24 (75.0)	32 (71.1)	0.765	47 (74.6)	9 (64.3)	0.679
Mild	6 (18.8)	8 (17.8)		11 (17.5)	3 (21.4)	
High	2 (6.3)	5 (11.1)		5 (7.9)	2 (14.3)	
Motivation rulers/ Importance						
0–3	6 (18.8)	4 (8.9)	0.174	8 (12.7)	2 (14.3)	0.738
4–7	14 (43.8)	15 (33.3)		25 (39.7)	4 (28.6)	
8–10	12 (37.5)	26 (57.8)		30 (47.6)	8 (57.1)	
Motivation rulers/confidence						
0–3	8 (25.0)	6 (13.3)	0.078	12 (19.0)	2 (14.3)	0.805
4–7	18 (56.3)	20 (44.4)		30 (47.6)	8 (57.1)	
8–10	6 (18.8)	19 (42.2)		21 (33.3)	4 (28.6)	
Motivation rulers/readiness						
0–3	8 (25.0)	12 (26.7)	0.036	16 (25.4)	4 (28.6)	0.969
4–7	17 (53.1)	12 (26.7)		24 (38.1)	5 (35.7)	
8–10	7 (21.9)	21 (46.7)		23 (36.5)	5 (35.7)	
The reasons for smoking cessation						
Recommendations from family members or others	6 (7.8)	5 (6.5)	0.289	10 (13.0)	1 (1.3)	0.835
For your own health	13 (16.9)	18 (23.4)		25 (32.5)	6 (7.8)	
Economic reasons	1 (1.3)	3 (3.9)		3 (3.9)	1 (1.3)	
Environmental reasons	0 (0.0)	1 (1.3)		1 (1.3)	0 (0.0)	
For clean image management	2 (2.6)	1 (1.3)		3 (3.9)	0 (0.0)	
For the health of the people around	5 (6.5)	4 (5.2)		8 (10.4)	1 (1.3)	
To show willingness to smoking abstinence	1 (1.3)	0 (0.0)		1 (1.3)	0 (0.0)	
Because of social gaze	1 (1.3)	0 (0.0)		1 (1.3)	0 (0.0)	
Etc	3 (3.9)	13 (16.9)		11 (14.3)	5 (6.5)	
The supporters for smoking cessation						
Parents/grandparents	8 (10.4)	8 (10.4)	0.387	15 (19.5)	1 (1.3)	0.512
Siblings	0 (0.0)	1 (1.3)		1 (1.3)	0 (0.0)	
Partner	1 (1.3)	5 (6.5)		5 (6.5)	1 (1.3)	
Children	1 (1.3)	4 (5.2)		3 (3.9)	2 (2.6)	
Friends/school peers	2 (2.6)	7 (9.1)		8 (10.4)	1 (1.3)	
Coworkers	0 (0.0)	0 (0.0)		0 (0.0)	0 (0.0)	
Teachers	9 (11.7)	6 (7.8)		13 (16.9)	2 (2.6)	
Healthcare professionals	0 (0.0)	0 (0.0)		0 (0.0)	0 (0.0)	
Others	3 (3.9)	5 (6.5)		5 (6.5)	3 (3.9)	
No one	8 (10.4)	9 (11.7)		13 (16.9)	4 (5.2)	

### Factors associated with smoking cessation maintenance

The number of counseling sessions attended was significantly associated with successful smoking cessation maintenance at 4 weeks (adjusted OR = 1.91, CI = 1.37–2.67) and 24 weeks (adjusted OR = 9.16, CI = 1.85–45.23). In particular, if the number of counseling sessions increased in 24 weeks, the chances of maintaining smoking cessation were 9.16 times higher. Pregnancy (adjusted OR = 8.03, CI = 1.94–33.23), CO levels (adjusted OR = 0.83, CI = 0.72–0.94), and drinking (adjusted OR = 0.14 and CI = 0.04–0.47) showed significant relevance to smoking cessation maintenance only at 4 weeks. Single mothers who were pregnant were 8.03 times more likely to quit smoking for 4 weeks, and those with higher CO levels that decreased were 0.83 times more likely to quit smoking for 4 weeks, and 0.14 times less likely to maintain smoking cessation for 4 weeks when drinking (Table 4).

**Table 4:** Factors associated with smoking abstinence according to quitting duration

Variable	4 weeks abstinence				24 weeks abstinence			
	Adjusted		Unadjusted		Adjusted		Unadjusted	
	Odds ratio (95% CI)	*p* value	Odds ratio (95% CI)	*p* value	Odds ratio (95% CI)	*p* value	Odds ratio (95% CI)	*p* value
Pregnancy	8.03 (1.94–33.23)	0.004	3.28 (1.06–10.14)	0.039	3.74 (0.75–18.68)	0.108	1.50 (0.11–5.13)	0.515
CO level	0.83 (0.72–0.94)	0.005	0.83 (0.73–0.94)	0.003	0.92 (0.78–1.10)	0.340	0.91 (0.78–1.07)	0.267
Number of counseling	1.91 (1.37–2.67)	0.000	1.75 (1.33–2.32)	0.000	9.16 (1.85–45.23)	0.007	3.65 (1.85–7.16)	0.000
Drinking	0.14 (0.04–0.47)	0.001	0.21 (0.08–0.57)	0.002	0.78 (0.19–3.19)	0.728	0.67 (0.19–2.35)	0.523

Adjusted by age and education level

## Discussion

In this study, pregnancy was found to increase the likelihood of short-term smoking cessation (4 weeks) in single mothers. Although women were likely to attempt smoking cessation during pregnancy [29, 30], over 60% of mothers who quit smoking during pregnancy relapsed within six months after childbirth. This phenomenon might occur because mothers only had external motivation to quit smoking, such as concerns for their children’s health, but lack internal motivation and, thus, only temporarily ceased smoking during pregnancy [31]. Adolescent mothers were also more likely to begin smoking again after cessation [32]. One study reported that pregnant adolescents aged 15– 19 years had the highest rate of smoking among all pregnant mothers [33]. In the present study, adolescent mothers who smoked accounted for 50% of all participants, indicating that adolescent mothers were more vulnerable to developing smoking habits. These results demonstrated that a smoking relapse prevention program tailored to single mothers and an intensive counseling program for adolescent mothers would be necessary to help single mothers maintain long-term smoking cessation, even after childbirth.

In this study, the likelihood of short-term smoking cessation maintenance increased as the participants’ exhaled CO levels decreased. This is consistent with a previous report that the rate of successful smoking cessation increased as exhaled CO level decreased in male university students [34]. The measurement of exhaled CO level is noninvasive, unlike blood or urine tests, and allows for immediate assessment in all clinical settings [35]. Immediately checking exhaled CO level also allows individuals to be aware of their smoking status and reinforces their motivation for smoking cessation.

This study showed that not drinking alcohol in single mothers is an important factor for maintaining short-term smoking cessation. This result was consistent with a previous report that smoking was strongly associated with drinking, and those who abuse or were dependent on alcohol would be more likely to smoke and had higher rates of tobacco consumption compared with the general population [36]. Another study reported that adult smokers who had high alcohol consumption levels or previously abused alcohol had higher nicotine dependence and poorer smoking cessation treatment outcomes [37]. While smoking and drinking were independent risk factors for cancer and cardiovascular disease, alcohol and cigarettes could interact when used together to drastically increase the risk of disease [38]. Smoking and alcohol use during pregnancy can significantly affect the neurological and cognitive function of the fetus, causing symptoms of Attention Deficit Hyperactivity Disorder (ADHD) as well as learning and memory deficits [39]. However, it has been reported that mothers vary in their levels of awareness about the risks of smoking and drinking. While mothers have been found to follow public health recommendations regarding smoking cessation during pregnancy and acknowledge the need to quit smoking, they are also reported to believe that alcohol consumption during pregnancy is safe as long as it is within a certain amount, due to the lack of clear, standard guidelines written by experts [40, 41]. These results suggest that counselors must emphasize the dangers of smoking and drinking during pregnancy, even in small amounts, through an education and counseling program, as well as recommend smoking cessation and alcohol abstinence.

Most smokers voluntarily attempt to cease smoking but fail to maintain their efforts. The mean likelihood of successfully quitting smoking within 6−12 months after the initiation of smoking cessation is 5 % or below [42, 43]. This indicates that it is difficult for smokers to quit on their own and that they require help from others to do so successfully. Such difficulties can be resolved through consistent counseling with an experienced smoking cessation counselor [44]. In this study, the most important factor in the short-term and long-term smoking cessation maintenance period was the number of consultations received by the single mothers. As the number of smoking cessation counseling sessions increased, participants were 1.91 times more likely to maintain smoking cessation in the short term and 9.16 times more likely to maintain smoking cessation in the long term. This result was consistent with a previous report that a smoking cessation program tailored to pregnant women could effectively promote smoking cessation [45, 46]. Counseling also plays an important role in enhancing motivation to maintain smoking cessation [34]. Motivational enhancement counseling teaches individuals that everyone can have the motivation to change and reinforces internal motivation by explaining in detail the steps required for making the changes necessary for smoking cessation [47].

In this study, the higher the readiness of the Motivation Ruler, the higher the probability of maintaining smoking cessation, which indicates that the motivation for smoking should be increased by emphasizing short-term smoking cessation counseling.

In this study, the most common reason to quit smoking was “for my health.” A smoking cessation counseling program focusing on single mothers’ health may effectively promote the maintenance of smoking cessation. It had previously been reported that counseling pregnant women briefly for 3−5 min during a medical examination and providing them with information about smoking increased the rate of smoking cessation by 30−70% [48, 49], suggesting that consistent and frequent smoking cessation counseling positively affected the maintenance of smoking cessation. In this study, most participants responded that they had no one to support their efforts to quit smoking, thereby further demonstrating the need for smoking cessation counselors. This finding suggests that counselors should provide high-quality counseling and fulfill their role in providing smoking cessation support, which was in line with a previous study reporting that receiving support from others increased the success rate of smoking cessation [50].

Incorporating the present findings in the existing curriculum for smoking cessation counseling for single mothers will enable single mothers to sustain smoking cessation. Factors that showed significant results in short-term smoking cessation maintenance (drinking, pregnancy, CO level, and number of counseling sessions) need to be emphasized in the first and third sessions of existing smoking cessation counseling programs. Single mothers should be provided with information on the correlation between drinking and smoking and actively supported to cease drinking and smoking together. For single mothers who are pregnant, it is necessary to provide information on the risks of smoking during pregnancy, how to quit smoking during pregnancy, and how to cope with smoking cessation. It is necessary to actively support single mothers through short-term smoking cessation counseling programs by informing them about the risks of CO inhalation, as well as to continuously monitor oneself during smoking cessation. The most important thing for single mothers to maintain long-term smoking cessation is to receive continuous counseling. Such counseling should focus on the psychological, social, and economic stress experienced by single mothers in the pregnancy and parenting process, and should actively utilize mothers’ internal strengths and social support to enable realistic countermeasures to smoking. Moreover, it is important for counseling to include sufficient discussion on how to cope with smoking cessation so that single mothers can apply such measures on their own after the consultation is over. In addition, the counseling skills of smoking cessation counselors will be important for the formation and implementation of a smoking cessation counseling curriculum for single mothers [51]. Counselors should be provided training to develop their counseling skills to enable efficient use of strategies and effective interventions for smoking cessation [52]. Successful implementation of a smoking cessation curriculum incorporating the current results will enable single mothers to successfully maintain smoking cessation.

This study has some limitations that should be noted. First, the results cannot be generalized, as the study only included single mothers who were smokers from a certain area of Seoul. Repeated follow-up studies involving single mothers from different regions across the country should be necessary. Second, as this study used secondary data, only the variables related to smoking cessation services could be examined. Third, the sample size was small due to the difficulty of finding and enrolling single mothers who were also smokers. The number of participants who maintained smoking cessation for 24 weeks was even smaller. Fourth, we did not recruit a control group to compare the effectiveness of the existing smoking cessation programs for single mother smokers.

However, despite these limitations, this study is the first to use data collected by a smoking cessation counselor who regularly visited a single mother facility for over six months. This study is meaningful in that it examined factors associated with smoking cessation maintenance in single mothers that were not sufficiently discussed in previous studies. The results of this study may be used as basic data for improving the quality of counseling provided by smoking cessation support services.

## Conclusions

Our results suggest that continuous smoking cessation counseling should be conducted to ensure effective long-term smoking cessation maintenance for single mothers. Visiting a smoking cessation service made smoking cessation easier for single mothers who did not know how to quit smoking through help from a smoking cessation counselor and allowed them to access smoking cessation services for a long period. To increase the smoking cessation rate of single mothers, it is important to conduct customized smoking cessation counseling. In the future, we recommend conducting additional follow-up studies on the health of smoking single mothers raising children.

## Data Availability

The datasets for the study are available from the corresponding author on reasonable request. The data was provided by KHPI (https://www.khealth.or.kr/kps).

## Abbreviations

CO: carbon monoxide
FTND: Fagerström Test for Nicotine Dependence
